# Robot-Assisted Total Proctocolectomy for Familial Adenomatous Polyposis with Multiple Colorectal Cancers Using the Hugo RAS System

**DOI:** 10.70352/scrj.cr.25-0035

**Published:** 2025-03-22

**Authors:** Yu Yoshida, Yuki Aisu, Yoshiro Itatani, Koya Hida, Ryosuke Okamura, Masahiro Maeda, Nobuaki Hoshino, Hisatsugu Maekawa, Atsushi Ikeda, Keiko Kasahara, Hiromitsu Kinoshita, Shigeo Hisamori, Shigeru Tsunoda, Kazutaka Obama

**Affiliations:** Department of Surgery, Graduate School of Medicine, Kyoto University, Kyoto, Kyoto, Japan

**Keywords:** robot-assisted TPC, Hugo RAS system, FAP

## Abstract

**INTRODUCTION:**

Experience with the Hugo RAS system in robot-assisted colorectal surgery is limited. This is particularly noticeable when focusing on complex procedures, such as total proctocolectomy (TPC). This study aimed to demonstrate the feasibility and safety of using the Hugo RAS system for TPC.

**CASE PRESENTATION:**

A 27-year-old woman with multiple colorectal cancers with a background of familial adenomatous polyposis underwent robot-assisted TPC, including lymph node dissection of the entire colorectal region using the Hugo RAS system. The robotic procedure was divided into 3 steps: 1) Trendelenburg position to perform ascending colon complete mesocolic excision (CME) to the hepatic flexure, 2) descending colon CME and total mesorectal excision with D3 lymph node dissection, and 3) flat position to perform central vessel ligation along the superior mesenteric artery. After undocking, the specimen was extracted transanally, and an ileal pouch was constructed from a small laparotomy at the umbilical incision, followed by ileal pouch-anal anastomosis. The operative time was 632 min, and the estimated blood loss was minimal. The postoperative period was uneventful.

**CONCLUSIONS:**

Robot-assisted TPC using the Hugo RAS system is safe and feasible. The flexibility of Hugo, which is carried by a modular-type surgical robot with multiple independent arms, enables safe and effective advanced procedures.

## Abbreviations

CME
: complete mesocolic excision
CT
: computed tomography
CVL
: central vessel ligation
FAP
: familial adenomatous polyposis
IMA
: inferior mesenteric artery
IMV
: inferior mesenteric vein
IPAA
: ileal pouch-anus anastomosis
MCA
: middle colic artery
POD
: postoperative day
SMA
: superior mesenteric artery
SMV
: superior mesenteric vein
TME
: total mesorectal excision
TPC
: total proctocolectomy

## INTRODUCTION

Robotic-assisted surgery has become the preferred approach for colorectal procedures because of its minimally invasive nature. It provides advantages, such as reduced postoperative pain, shorter hospital stays, and faster recovery than traditional open surgery.^1–3)^ The Hugo RAS system is an innovative surgical robot designed to enhance operational flexibility, as it is a modular-type surgical robot with multiple independent arms mounted onto independent surgical carts. In addition, it also offers a wide range of joint motion, which enables us to perform upper to lower abdominal surgery from the same trocars (**Supplementary Fig. S1**).

To date, experience with the Hugo RAS system in colorectal surgeries has been limited, with most reports focusing on major surgeries, such as right or left hemicolectomies and rectal anterior resection.^4)^ The application of Hugo for total proctocolectomy (TPC), especially with comprehensive lymphadenectomy across multiple regions of the colorectum, presents unique technical challenges that require precise coordination of the robotic arms for optimal access and control across different abdominal quadrants from the upper side to the lower pelvic floor.

Here, we present the first case of robot-assisted TPC with D3 lymphadenectomy across the entire colorectum using the Hugo RAS system in a patient with familial adenomatous polyposis (FAP) complicated by multiple colorectal cancers. This case demonstrates the safety and feasibility of multi-quadrant colorectal surgery using Hugo.

## CASE PRESENTATION

A 27-year-old woman with multiple colorectal cancers on a background of FAP was presented to our department. Notably, a large lesion was detected in the ascending, transverse, and sigmoid colon and the upper rectum, and pathological examination confirmed some of them as adenocarcinoma. Preoperative computed tomography revealed multiple lymph node swellings along the inferior mesenteric artery (IMA) and middle colic artery, without any evidence of distant metastases. After a comprehensive evaluation by a multidisciplinary cancer board, we decided to perform TPC with lymph node dissection of the entire colorectal region, using the Hugo RAS system as a surgical device.

Robot-assisted TPC using the Hugo RAS system was approved by the Evaluating Committee for Highly Difficult New Medical Technologies (approval number H-0051) and the Institutional Review Board at Kyoto University.

**Fig. 1:**
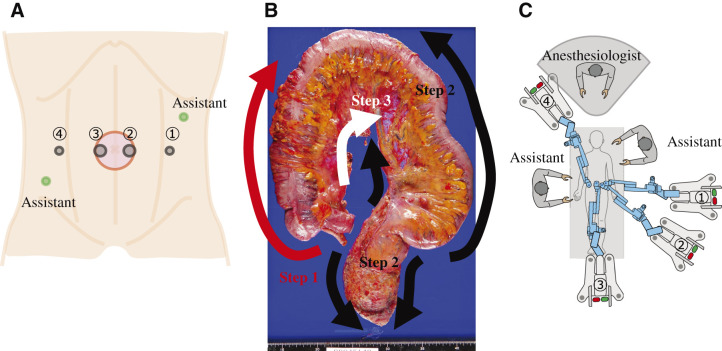
Scheme of the port placement, procedure steps, and arm cart layout. (**A**) Trocar layout for robot-assisted TPC. Four robotic trocars were used. Two assistant trocars were placed on the upper left and lower right of the abdomen. (**B**) Three steps of the surgical procedure. The robot-assisted TPC procedure was divided into 3 steps. The surgical procedure was successfully completed using 2 Hugo dockings and patient repositioning. (**C**) The operating room setup of the robot-assisted TPC. TPC, total proctocolectomy

Under general anesthesia, the patient was placed in a lithotomy position with the arms tucked. After a 5-cm vertical skin incision was made at the umbilicus, a wound-protecting device was applied. After pneumoperitoneum, 4 robotic trocars and 2 assistant trocars were placed (**Fig. 1A**). The instruments used in robot-assisted TPC with Hugo were a camera, monopolar curved shears for the right hand, bipolar fenestrated forceps for the left hand, and Cadiere/double fenestrated forceps for the reserve arm (**Table 1**). Robot-assisted TPC with Hugo consists of 3 distinct steps (**Fig. 1B**), followed by transanal specimen extraction, ileal pouch construction through a small laparotomy, and ileal pouch-anus anastomosis (IPAA). Two table positions, Trendelenburg and flat, were required, each with specific docking tilts but the same angles of the arm carts throughout the robotic procedure. The detailed operative procedure is presented in **Supplementary Videos**. Details of the docking angles, tilts, and instruments of the robotic arm at each step are presented in **Figs. 1A** and **1C**, and **Table 1**.

**Table 1:** Docking angles and tilt settings at each surgical step for robot-assisted TPC

	Table position	Arm	Role	Instrument	Docking angle	Tilt
Step 1	Trendelenburg	1	Reserve	Cadiere forceps	90	15
		2	Right hand	Monopolar curved shears	135	−5
		3	Camera	30° oblique	180	−20
		4	Left hand	Bipolar fenestrated forceps	320	30
Step 2	Trendelenburg	1	Reserve	Cadiere forceps	90	15
		2	Left hand	Bipolar fenestrated forceps	135	−5
		3	Camera	30° oblique	180	−20
		4	Right hand	Monopolar curved shears	320	30
Step 3	Flat	1	Right hand	Monopolar curved shears	110	15
		2	Camera	30° oblique	140	0
		3	Left hand	Bipolar fenestrated forceps	195	15
		4	Reserve	Cadiere forceps	320	−30

TPC, total proctocolectomy

*Step 1*: Ascending colon complete mesocolic excision (CME)

The ascending colon CME from the caudal approach proceeded until the completion of the hepatic flexure mobilization (**Supplementary Video S1**).

*Step 2*: Central vessel ligation (CVL) of the IMA, descending colon CME, and total mesorectal excision (TME)

After CVL of the IMA, descending colon CME proceeded until the completion of splenic flexure mobilization, followed by TME until the intersphincteric space was fully exposed (**Supplementary Video S2**).

*Step 3*: CVL along the superior mesenteric artery (SMA)

After undocking all the robotic arms, the patient was placed in a flat position. Then, CVL along the SMA was performed to ligate the ileocolic, right colic, and middle colic vessels (**Supplementary Video S3**). The final step of this procedure was the ligation of the inferior mesenteric vein (IMV) at its root, which was exposed in Step 2.

### Transanal and small laparotomy procedures

After transection of the terminal ileum, we extracted the specimen transanally by excising the rectal mucosa entirely from just below the dentate line because of multiple adenomas in the anal canal. After constructing the ileal pouch through the small umbilical incision and confirming that the ileal pouch could reach the bottom of the anal canal for anastomosis, transanal hand-sewn IPAA was performed. A diverting ileostomy was not performed.

**Fig. 2:**
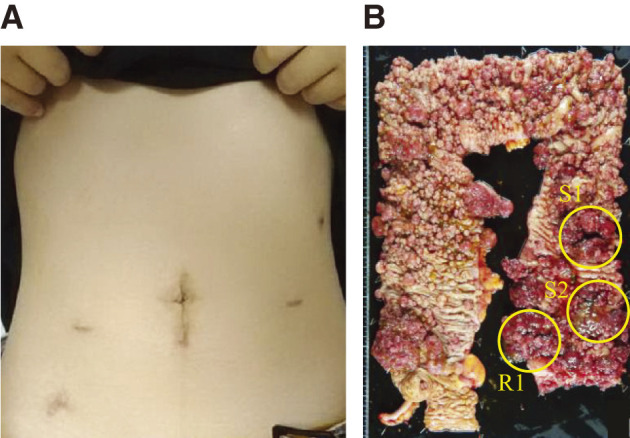
Postoperative findings. (**A**) Abdominal aspect at 3 months after surgery. (**B**) Surgical specimen. Yellow circles indicate cancer lesions.

All 3 steps were completed without conversion to open surgery. After undocking Hugo when we finished Step 3, we performed a laparoscopy to confirm hemostasis, specimen extraction, and appropriate anastomosis. The operative time was 632 min (36 min for Step 1, 160 min for Step 2, 188 min for Step 3, and 248 min for other procedures such as positioning, docking, specimen extraction, and anastomosis), with a minimal intraoperative estimated blood loss of 20 mL. The patient exhibited an uneventful postoperative recovery, with gas passage and initiation of liquid nutrition on postoperative day 1 (POD 1) and a solid diet on POD 3 with a functional ileal pouch and satisfactory anal function. A photograph of the abdomen taken 3 months postoperatively is shown in **Fig. 2A**. Pathological examination revealed 2 sigmoid colon cancers (S1, Type 0-Ip, 55 × 50 mm, tub1, T1b, ly0, v0; S2, Type 0-Isp, 55 × 50 mm, tub1, Tis, ly0, v0) and 1 rectal cancer (R1, Type 0-Ip, 40 × 35 mm, tub1, Tis, ly0, v0). It also revealed 18 out of 89 positive lymph nodes, all of which belonged to the sigmoid colon and rectosigmoid lesions (stations #241, 242, and 251), resulting in UICC pT1bN2b stage (**Fig. 2B**).

## DISCUSSION

Robot-assisted surgery has become a standard approach in several surgical fields worldwide.^5–8)^ We performed robot-assisted TPC using the Hugo RAS system for the first time. Total console time was 384 min, which was acceptable because this case required D3 lymphadenectomy across the entire colorectum. However, the non-console time was 248 min, which can be improved by repeating this procedure as a team.

The Hugo RAS system is one of the newly launched robotic systems. It features a modular-type robotic system with 4 independent arms mounted onto independent surgical carts. They allow configuration freedom by considering both the surgeon’s preference and patient characteristics. Therefore, proper arm positioning and docking angles are critical to ensure procedural feasibility and efficiency because they help prevent arm collisions and facilitate access to multiple abdominal quadrants.^9)^ Moreover, the range of the arm joint is expansive, reaching 180° when combined with tilt, which allows surgeons to complete surgeries with extended surgical fields, such as TPC (**Supplementary Fig. S1**). This case requires operations in the deep pelvis and lymph node dissection along SMA/superior mesenteric vein (SMV). Also, it is often difficult to proceed with robot-assisted TPC in the splenic flexure, the boundary between the surgical areas centered on the right colon and the rectum due to limitations in arm movement. In such cases, proper patient positioning with a wide range of joint motion of the Hugo arms helps precise procedures. The open surgeon console also improves team communication and enables observers (e.g., trainees) to follow the operation using 3-dimensional visualization. Pistol-like grips, which resemble those of laparoscopic instruments, also offer high compatibility to laparoscopic surgeons without prior surgical robot experience.^10,11)^

A limitation of the Hugo RAS system in major colorectal surgeries is the absence of wristed robotic advanced energy devices, clip appliers, and wristed linear staplers.^11–13)^ In this case, the console surgeon only used monopolar curved shears for lymphadenectomy, and the patient-side assistant clipped the vessels for CVL. Some TPC cases, such as FAP with only adenomas or ulcerative colitis, do not need lymphadenectomy across the entire colon. In such cases, advanced energy devices are essential to dissect along the colorectum for efficient and speedy dissection.

To the best of our knowledge, this is the first reported case of robot-assisted TPC, including lymph node dissection of the entire colorectal region, performed using Hugo. Previous reports of robot-assisted TPC using da Vinci Xi showed similar port placement.^14–16)^ Da Vinci Xi requires boom rotation when switching between upper and lower abdominal procedures, whereas Hugo requires tilt adjustment when changing patient position, which is the difference between boom- and modular-type surgical robots. In Japan, colorectal cancer surgery using the Hugo RAS system has gradually become widespread. Even for standard procedures such as right hemicolectomy or rectal anterior resection, optimal trocar placement remains a topic of debate.^4,17)^ Moreover, performing TPC with lymph node dissection across the entire colorectal region adds further complexity. We considered TPC to be a combination of standard right hemicolectomy and rectal anterior resection. During Step 3, minor arm interference was encountered while mobilizing the transverse colon and approaching the middle colic vessels. However, the procedure was completed without any complications, and CME, CVL, and TME were achieved with Hugo.

Robot-assisted surgery for colorectal cancer is a promising procedure that produces better short- and long-term outcomes than conventional laparoscopy.^1–3,5)^ However, it is reported that robotic surgery requires higher costs than conventional laparoscopy.^18)^ Therefore, to facilitate the widespread use of robotic surgery, it is important to lower these costs. The unit prices of Hugo devices are lower than those of other robotic systems (**Supplementary Table S1**), which may contribute to their widespread use. The patient had good perioperative recovery without any postoperative complications. The patient’s short-term quality of life and psychological status were high, with no diverting stoma or urinary incontinence. Therefore, we can say that robot-assisted TPC using Hugo is of high quality.

## CONCLUSIONS

We report the first case of robot-assisted TPC, including lymph node dissection of the entire colorectal region, for FAP with multiple colorectal cancers using the Hugo RAS system, achieving safe and oncologically appropriate surgery. The Hugo RAS system represents an emerging minimally invasive robotic platform capable of facilitating procedures, such as TPC as well as standard colorectal surgeries.

## SUPPLEMENTARY MATERIALS

Supplementary Fig. S1 Joint movement of the robotic arm. The upper panel shows the maximum extension of the arm with a negative tilt, which enables it to reach a flat position to the right. The lower panel shows the minimum contraction of the arm with a positive tilt, which enables it to reach a flat position to the left.

Supplementary Table S1 Original unit prices (JPY).

### Supplementary Videos

Robot-assisted total proctocolectomy using the Hugo RAS system.

Supplementary Video S1 Step 1: Retroperitoneal approach for ascending colon CME until hepatic flexure mobilization.

Supplementary Video S2 Step 2: Descending colon CME and TME. Following IMA ligation, dissection proceeds toward the IMV through the omental bursa. TME is then performed, advancing from the retrorectal space to the intersphincteric plane.

Supplementary Video S3 Step 3: After repositioning the patient, CVL along the SMA and SMV was performed, including sequential ligation of the ileocolic, right colic, and middle colic vessels, and IMV.

## DECLARATIONS

### Funding

No funding was received for this study.

### Authors’ contributions

YY conceived and designed the study and drafted the manuscript.

YA, YI, KH, RO, MM, NH, HM, AI, KK, HK, SH, ST, and KO critically revised the article and approved the final version for publication.

All authors have read and approved the manuscript.

### Availability of data and materials

All data supporting the conclusions of this study are included in the published article.

### Ethics approval and consent to participate

Patient privacy was considered, and the manuscript includes no identifying information. Our institution does not require ethical approval for case reports.

### Consent for publication

The patient provided informed consent for the publication of this case.

### Competing interests

The authors declare the following potential conflict of interest: Kazutaka Obama and Koya Hida have received honoraria from Medtronic. The other authors declare that they have no competing interests.
