# Supplementary figures and images for: Robot-Assisted Total Proctocolectomy for Familial Adenomatous Polyposis with Multiple Colorectal Cancers Using the Hugo RAS System
Source: Surg Case Rep. 2025 Mar 22;11(1):25-0035. doi: 10.70352/scrj.cr.25-0035 (PMC11946455; doi:10.70352/scrj.cr.25-0035)

Supplementary Fig. S1

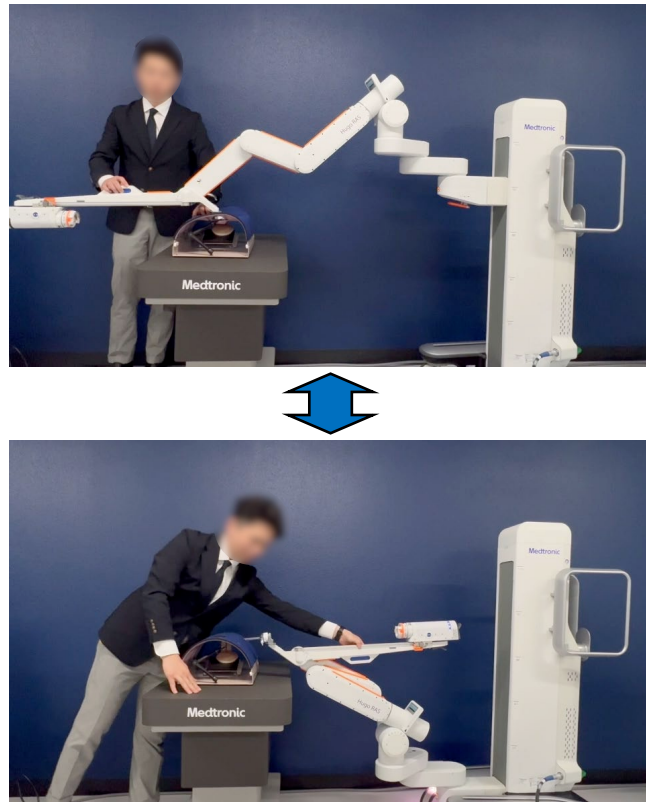

Supplement: Supplementary Fig. S1 [file scr-11-01-25-0035-s001.pdf]
