# Supplementary material for: Robot-Assisted Total Proctocolectomy for Familial Adenomatous Polyposis with Multiple Colorectal Cancers Using the Hugo RAS System
Source: Surg Case Rep. 2025 Mar 22;11(1):25-0035. doi: 10.70352/scrj.cr.25-0035 (PMC11946455; doi:10.70352/scrj.cr.25-0035)
Supplement: Supplementary Table S1 [file scr-11-01-25-0035-s002.pdf]

Supplementary Table S1

Original unit prices (JPY)

		Hugo™ RAS system	da Vinci Xi	hinotori™
Monopolar curved shears<br>Monopolar curved scissors	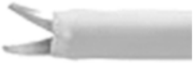	21,800	41,130	38,800
Bipolar fenestrated grasper<br>Fenestrated bipolar<br>Bipolar fenestrate	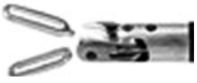	21,800	29,686	28,000
Cadiere forceps<br>Cadiere<br>Universal grasper	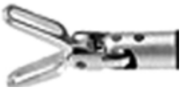	18,500	22,261	21,000
Total		62,100	93,077	87,800
